# Behavioral Training in First-Generation Long-Tailed Macaques (*Macaca fascicularis*) for Improved Husbandry and Veterinary Procedures

**DOI:** 10.3390/ani14162369

**Published:** 2024-08-15

**Authors:** Lalitta Suriya-Arunroj, Motee Chimngam, Chutikan Chamnongpakdee, Thipchompoo Sing-Ayudthaya, Chunapa Linchekhaw, Nopparat Kongsombat, Nutchanat Suttisan

**Affiliations:** National Primate Research Center of Thailand, Chulalongkorn University, Saraburi 18110, Thailand

**Keywords:** long-tailed monkeys, distress, saliva collection, cortisol, venipuncture, primate chair

## Abstract

**Simple Summary:**

Behavioral training is an internationally accepted and recommended tool in enhancing the welfare of captive animals, facilitating their habituation to unfamiliar environments, and promoting cooperation with routine care procedures. Long-tailed monkeys were previously reported to be fearful and difficult to train. Moreover, wild long-tailed monkeys from monkey–human conflict areas can add more challenges; their prior experience with humans can either facilitate or complicate training processes. This project aimed to apply behavioral training techniques, established for captive-born primates, to test whether wild long-tailed monkeys could be trained to cooperate with routine and animal-care procedures and to determine the time required for each training exercise. Additionally, cortisol levels, a key indicator of stress, were monitored to evaluate the animals’ stress responses, providing an additional metric for determining the success of training. This study offers the first validation of a primate behavioral training program wild long-tailed macaques, contributing valuable insights into the refinement of welfare practices for this specific population.

**Abstract:**

Owing to their similarities to humans in various aspects, non-human primates (NHPs) serve as valuable translational models that has greatly contributed to scientific advancements. However, working with untrained NHPs can cause stress and increase the risk of injuries to both animals and care staff, compromising both animal welfare and occupational safety. Behavioral training, that benefits from animals’ learning abilities to gain their cooperation during husbandry and veterinary procedures, is a well-established method to mitigate these risks. Cynomolgus monkeys, in particular, are known for being despotic, fearful, and challenging to train. Moreover, our first-generation breeders were wild-sourced from human–macaque conflict areas in Thailand. These macaque populations are accustomed with human contact; hence, their prior experience can either work for or against behavioral shaping plans. Establishing a training program with realistic expectations would benefit both the animals and trainers. In this study, six cynomolgus monkeys were selected based on temperament, then underwent a pilot training program that included basic husbandry and veterinary procedures. Over 256 training sessions with gradual shaping plans, all six monkeys went through all training steps, with progress varying considerably among individuals. Cortisol levels were measured to monitor stress responses, revealing a notable sex difference: female monkeys generally complied more easily with the trainer but exhibited a stronger cortisol increase compared to males. This study proposed a behavioral training program grounded in three essential components: temperament assessment, behavioral shaping plans, and the cortisol-based criteria for evaluating training success.

## 1. Introduction

On the basis of their genetic and physiological relatedness to humans, nonhuman primates (NHPs) share multiple common features with humans, and the data obtained from NHP models provide greater validity compared to other animal models [1,2,3,4,5]. Further, certain scientific questions can only be addressed using NHP models and no other species (e.g., models of AIDS, lung disorders, drug metabolism, and neurosciences) [2,3,4,5,6,7,8,9,10]. NHPs are thus essential translational models that have greatly contributed to advances in biomedical research, enabling the exploration, understanding, and the development of prevention and treatments for numerous conditions that inflict great suffering on both humans and animals [1,5,6,9,11,12,13,14,15,16].

However, the close similarity of NHPs to humans, especially their highly developed brains and complex social behaviors, raises specific ethical questions and practical challenges in terms of meeting their behavioral, environmental, and social needs in captive environments [17,18,19,20,21,22,23,24,25,26,27,28]. NHPs are intelligent (capable of experiencing boredom and depression in an unchanging, monotonous environment [20,25,29,30,31,32,33]), social (requiring compatible conspecific or human companionship for their emotional well-being [17,19,34,35,36]), sensitive (prone to intense fear when forcibly restrained [37,38,39,40]), and undomesticated (not naturally tame, even when bred in captivity [37,38,40]). Hence, solely providing shelter and food is insufficient to support their well-being; therefore, special care must be taken for both physical and mental health.

Explicit and enforceable rules now go beyond the basic requirements of adequate food, housing, and veterinary care to address the cognitive and psychological complexity of NHPs [5,18,41,42,43,44], including social housing, environmental enrichment, and positive reinforcement training (PRT). In 1985, the U.S. Animal Welfare Act mandated that facilities housing NHPs must provide for their psychological well-being. Since then, there has been an increased interest from regulatory and accrediting agencies to ensure improved conditions for captive NHPs [17,18,23,27,28,45,46]. PRT has become a recognizable component of progressive captive animal management, and every facility caring for NHPs should integrate training strategies into its management systems and be equipped with specialized training in animal handling, capture, enrichment, biohazard compliance, and the experience necessary to work with NHPs [47,48,49,50,51,52,53,54,55,56].

Pain and fear are the primary experiential phenomena underlying stress in multiple species of laboratory animals, and the animals may not spontaneously habituate to husbandry and research-related procedures, such as home cage cleaning, transfers, social-housing arrangements, unfamiliar personnel entering an animal room, or environmental disturbances in the laboratory [38,40,57,58]. Without training, any major unfamiliar stimuli or sudden forced contact with humans can cause panic to the naïve animals. Fear responses (e.g., freezing, avoiding, or aggressing) toward humans are considered an indicator of stress and poor captive welfare [37,58,59,60]. Behavioral training techniques can reduce fear responses and enhance welfare [48,52,54,56,61,62,63]. Specifically, NHPs were traditionally perceived as challenging and dangerous to handle, necessitating restraint during veterinary and experimental procedures to protect both investigators and animals. However, forceful restraint can spontaneously exacerbate fear and defensive behaviors, turning such procedures more dangerous [37,50,56,64,65]. Working with untrained animals thus causes stress and potential injuries to both animals and care staff, thus jeopardizing both animal welfare and occupational safety.

Positive reinforcement training (PRT) is a systematic training method that originated in marine mammal parks for training show animals in front of an audience, later evolving to encompass husbandry procedures, including veterinary access to collaborating unanesthetized animals. Nowadays, the applications and range of genera have vastly expanded [66,67,68,69,70]. Positive reinforcement occurs when a desired behavior is immediately rewarded, making that behavior more likely to be repeated in the future [48,52,67,71,72]. Through PRT, the animal can learn to voluntarily cooperate in husbandry and veterinary procedures, rather than having to submit to chemical (anesthesia) or physical restraint [48,49,50,54,56,68]. Working with cooperative animals is safer and less stressful for researchers, veterinarians, animal care staff, and the animals. In addition to animal welfare and occupational health considerations, chronic stress can alter the physiology, brain structure, and brain function [58,60,73,74,75], confound some types of research studies [76,77,78], and compromise the quality of study results [3], thus jeopardizing the objectives of animal research [5,28,36,44,79]. Taken together, behavioral training helps alleviate the adverse consequences of captivity by reducing animals’ fear and anxiety from restraint, reducing the use of anesthesia, increasing animals’ sense of control, improving animal–human interaction, rendering veterinary and experimental procedures less time- and effort-consuming, and, last but not least, enhancing the reliability of experimental data [45,48,51,53,54,55,56,68,80,81,82].

To complying with internationally agreed animal-use standards and support animal welfare, NPRCT-CU is establishing a behavioral training program to alleviate animals’ stress and gain cooperation from NHPs. The first-generation cynomolgus macaque breeders at the facility were sourced from human–macaque conflict areas in Thailand. In contrast to captive-bred animals, these wild-sourced animals experience stress from capture and transport [37,40,83,84], abrupt changes from their habitat to captivity, including unfamiliar environments, human presence, and other potential stressors [38,57,65], which may impose more difficult challenges on them. In particular, this macaque population had prior human experience, which may either support or oppose standard behavioral shaping plans. Furthermore, long-tailed macaques or crab-eaters (cynomolgus; *Macaca fascicularis*) have been reported to be fearful and difficult to train and display higher levels of fear responses than rhesus (*Macaca mulatta*) and bonnet macaques (*Macaca radiata*) [85,86,87]. Establishing a training program based on previous studies in captive-born macaques may lead to unrealistic expectations of animal progress and may render training procedures too stressful for both staff and animals.

This study aimed to establish a behavioral training program for first-generation long-tailed macaques with prior human exposure, estimating the duration needed for each training step and setting reasonable milestones for this specific NHP population. The program was based on PRT programs from previous reports [26,45,47,48,49,51,52,53,54,56,70,79,81,82,88,89,90,91,92], integrating with procedures that care and veterinary staff considered helpful for husbandry, veterinary, and potential experimental procedures, e.g., voluntary venipuncture, chewing on a baited rope for saliva collection, and chair training. This project was an initial step toward a long-term PRT program for both animal and staff training, enhancing training skills for animal-care staff [26,45,53,79,88], increasing awareness of the benefits of behavioral training and animal welfare concerns for research animals, and paving the way for more sophisticated research, such as behavioral and cognitive neuroscience research, which requires complex behavioral reports [7,76,77,78,93,94]. Additionally, behavioral training techniques developed for wild-sourced animals in this study can be applied to captive and wild-sourced animals in other contexts, such as zoos, shelters, sanctuaries [33,37,95,96,97], as well as to manage strayed macaques in countries where human–macaque conflict remains a challenge.

## 2. Materials and Methods

### 2.1. Animal Subjects

Six long-tailed macaques (*Macaca fascicularis*), comprising 3 males and 3 females, aged 3–6 years (mean ± SD: 4.0 ± 1.4 years of age) and weighing 2.89–3.99 kg (mean ± SD: 3.49 ± 0.43 kg) were selected for participation in a behavioral training program (see details below). Each individual was identified by a unique name and number; none had been exposed to any formalized animal training program prior to this study.

The monkeys were initially housed in social-housing cages at the breeding facility (4 m deep × 4 m wide × 2.5 m high). After six animals were selected for individualized training (see ‘*Shaping plans*’ below), they were transferred and grouped into two same-sex groups of three, housed in a quadrant cage (190 cm high × 125 cm wide × 70 cm deep; each section measured 85 cm high × 60 cm wide × 70 cm deep) situated in a semi-opened environmental system in the breeding facilities at the National Primate Research Center of Thailand, Chulalongkorn University (NPRCT-CU, Saraburi, Thailand). The opened buildings of the breeding facilities had high ceilings with heat-insulated roofs equipped with electric fans to increase ventilation. The relative humidity, under natural conditions, averaged 68 ± 12 (mean ± SD; ranging between 29 and 94% RH), and the temperature measured 28.5 ± 3.4 (mean ± SD; ranging between 17.1 and 37.8 °C). The lighting conditions followed natural conditions, which did not significantly differ during the entire year in central Thailand. The average sunrise and sunset times were 06:12 and 18:23, respectively. Electric lighting systems were installed and could be switched on between 8:00 and 17:00. The quadrant cages were equipped with divider panels to allow temporary separation between the animals during the training periods when concentration and no interruption from peers were required. The animals were fed standard monkey chow (Perfect Companion Group Co., Ltd., Bangkok, Thailand) in the mornings (09:00–10:00) and fresh produce in the afternoons (14:30–15:30). Fresh drinking water was available ad libitum. The NPRCT-CU animal care program was accredited by the Association for Assessment and Accreditation of Laboratory Animal Care (AAALAC)—International, and all animal procedures were evaluated and approved by the NPRCT-CU Institutional Animal Care and Use Committee (IACUC) under protocol number 2075011.

### 2.2. Animal Selection

Prior to individualized training, the trainer initially interacted with approximately 30 cynomolgus macaques (aged 2–17 years) housed in groups of up to 15 animals in gang cages (large social-housing cages in the NPRCT-CU breeding facility; 4 m deep × 4 m wide × 2.5 m high) to train them in basic exercises (Step 1: *habituation*). No individual was separated from their groups during the initial interactions; therefore, habituation was conducted within the constraints imposed by the social hierarchy of the groups. Because temperament is a crucial factor affecting training efficacy [98,99,100,101], the trainer assessed the animals’ temperament while interacting with the animals in the gang cages by checking the animals’ motivation (whether the animal accepted and consumed treats in the trainer’s presence), confidence (whether the animal approached and remained near the trainer at the cage front), curiosity (whether the animal interacted with an unfamiliar object presented at the cage front), and trainability (whether the animal demonstrated learning of the target behavior during training). The first six animals meeting all four criteria were selected from the colonies for subsequent individual training. The selected animals that entered the individualized training program (from Step 3: *saliva collection training* onwards) were transferred into quadrant cages and remained in same-sex social groups of three. During individual training, the animals were separated from their cage mates while maintaining visual and auditory contact. The animals were trained in varying order across days and returned to their social groups immediately after the training session.

### 2.3. Training Session

The macaques underwent training sessions up to 5 times per week (average: 3.3 ± 1.3 (mean ± std) days/week; range: 1–5 days/week). Each training session lasted an average of 8.27 ± 4.01 (mean ± std) minutes/day, ranging from 2 to 15 min/day, depending on the animal’s engagement, progress, and satiation levels, except for the serial-blood-collection training using an IV catheter, which lasted up to 2 h (see Step 7: *Serial Blood Collection*). Training was consistently scheduled between 9:00 and 12:00 to maintain optimal levels of alertness and comparable levels of the cortisol hormone across sessions. Training durations and reinforcements (see *Primary* and *Secondary reinforcers* in Supplementary Methods) were regularly adjusted to ensure that the animals’ attention and engagement were high throughout training. Once the animals showed good progress, the duration of the training sessions was gradually increased. Based on previous research, one training session per day was determined to be the most efficient frequency for macaque training [81,102].

### 2.4. Shaping Plans

Animals were trained by reinforcing successive approximations of the target behaviors [59]. Shaping involved breaking down the desired behavior into small incremental steps and then teaching one step at a time until the desired behavior was achieved [51,53,88,89]. If, at any point, the animal failed to maintain the learned steps or advance to the next step, the trainer reverted to the previous stage to reinforce learned behaviors before progressing. Sessions often began at a slightly lower level than previously achieved to reinforce the repetition of learned behaviors and to motivate the animals before gradually moving to the next training step. For this reason, each session was typically completed with a short series of successes at the most recently achieved level of the animal.

According to [48,52,53,54,88,89], we constructed a shaping plan for each desired behavior as follows (see Training Details in Supplementary Methods):

1. Habituation: reinforcer bridging, hand feeding, and syringe feeding

**Figure 1:**
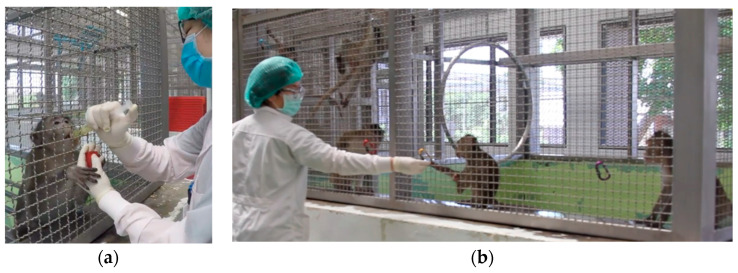
(**a**) Target and (**b**) station training.

Habituation was conducted with socially housed animals to habituate them with the trainer and the clicker device [72,82,91,102,103]. The trainer fed the animals (primary reinforcer) while sounding the clicker device (secondary reinforcer) to bridge the clicker sound, which later became a rewarding signal (reinforcer bridging) [48,53,56,66,71,72,81,82,88,89,102,103,104]. Then, the trainer familiarized the animals with hand and syringe feeding (Figure 1a). Syringe feeding was a training step that benefited trainer–animal interaction, veterinary procedures, such as drug administration via the oral route. It also allowed the animals to become accustomed to the presentation of syringes, which helped to facilitate needle training in the later training steps. This initial interaction also allowed the trainer to assess the animals’ motivation, confidence, and curiosity as part of temperament screening.

2. Target and station training (adapted from [30,47,53,54,56,81,82,88,105])

Target and station training were also conducted for socially grouped animals. Target training is an initial step in establishing learning rules, and it provides an important foundation for various behavioral training possibilities. The objective of target training was to instruct the animal to touch its body parts to a moveable object (e.g., a handheld carabiner). The achievement of target training by an animal was also one of the temperament criteria, i.e., trainability, used for selecting animals for further training. 

In social cages with multiple animals, dominant animals were typically the first to interact with the trainer and often attempted to monopolize training. Station training allows access to lower-ranked animals and reduces the likelihood that dominant animals will interrupt training sessions to steal treats. The goal of station training was to instruct the animal to move to and remain at a specific location or next to a stationary object (carabiners attached at the different locations at the enclosure front: Figure 1b). Once the more confident individuals started holding the target, station training could be introduced to allow access to subordinate individuals.

3. Saliva collection using baited ropes

Baited ropes, polyester ropes soaked in sugar solution and oven-dried, are commonly used for saliva sample collection in awake monkeys [106,107,108]. The baited rope was attached to a carabiner for ease of handling and collection. The clicker device was sounded when the animal chewed on the rope.

4. Presenting a leg for venipuncture (needle training; adapted from [49,50,53,61,62,63,64,65,68,88,89,104,109])

Needle training desensitizes injection and blood collection processes and provides an opportunity for animals to voluntarily cooperate with one of the most common care- and research-related procedures. The shaping plan started with foot target training; the trainer encouraged the animals to touch a target with their feet. After the animals were accustomed to having their ankle wrapped in the trainer’s hand for a few seconds, the needle training was conducted by desensitizing the animal to the venipuncture procedure with capped needles. If the animal remained calm throughout, the trainer applied the use of real needles.

5. Collar-pole training (adapted from [110,111,112])

Before the start of this step, the animals were anesthetized and a collar (Unifab Corporation, Portage, MI, USA) was loosely fitted around the animal’s neck. After collar placement, the animals were returned to their home cage and no further procedures were performed for a few days to allow for their habituation to the collar. The shaping plan of collar-pole training started with collar-pole touch: the trainer encouraged the animal to approach and lightly tapped the pole on the animal’s collar, then the animal was rewarded. Upon animal responses, the trainer extended the collar-pole touch time while encouraging the animal to remain calm. If the animal remained calm, the trainer hooked the collar using the pole eyelet and gently guided the animal out of the home cage.

6. Chair training (adapted from [64,94,111,112,113,114])

Chair training can help facilitate short-distance animal transfer without anesthesia, as well as enable access to the animal’s body parts for veterinary procedures. After the animal had mastered collar-pole training, the trainer guided the animal to a primate chair where they were offered food rewards. Then, the animal underwent approximations toward calmly sitting in the primate chair. The trainer secured all the chair parts, and the chaired animal could be wheeled out of the home cage area for a few minutes.

7. Serial blood collection using an IV catheter

Serial blood collection using intravenous catheters allows the collection of multiple blood samples in a short period while preventing blood vessel collapse, which is potentially caused by multiple needle-and-syringe venipunctures. Serial blood collection can facilitate veterinary procedures, such as blood gas monitoring, blood glucose monitoring, coagulation test, endocrine function test, etc. The aimed chair time and blood sampling time points were adapted from standard glucose tolerance tests to monitor the time course of cortisol responses throughout the chairing period in the current study. This protocol can potentially be applied for diabetic management. 

### 2.5. Performance Criteria

A behavior was considered learned when a subject could consistently and confidently repeat it successfully 20 times (for most exercises, except venipunctures) in one session without exhibiting fearful behavior, e.g., vocalizations such as alarm peeps, cackling and yapping, nervous grabbing/pulling, excessive hand rubbing, self-scratching, evading to the back of the cage, acute diarrhea, or attempts to bite/scratch trainer [82]. Regarding the training steps that involved venipuncture, the exercise was considered successful when the animal calmly accepted venipunctures 3 times.

### 2.6. Documentation of Training Sessions

To monitor training progress, we recorded information about each training session, including the session duration, the highest training step reached for each session, and whether negative reinforcement (e.g., the squeeze mechanism recessed upon desired behavior) or other techniques were used [111]. For each training step, we measured the time required and the behavioral effects of the training procedures to assess training effectiveness [51,81].

### 2.7. Cortisol Assays

Blood samples were collected during Step 4 (*present leg for venipuncture*) and Step 7 (*serial blood collection using an IV catheter*). A maximum volume of 1 mL per sample was immediately transferred into a lithium heparin tube, and kept on ice throughout the training session. After leaving the animal facility, the heparinized blood samples were centrifuged at 1000× *g* at 4 °C for 10 min. The resulting plasma was transferred into a 1.5 mL microcentrifuge tube, then either assayed immediately or stored at −20 °C until assayed. Plasma cortisol levels were measured using VIDAS^®^ Cortisol S (CAT number: 30451, Biomérieux, Marcy-l’Étoile, France). The accuracy of cortisol quantification using VIDAS^®^ Cortisol S was validated by comparing the values against monkey-specific cortisol standards (Cusabio, Houston, TX, US). The correlation coefficient between the VIDAS^®^ assay and the ELISA method was found to be 0.9920, indicating a high degree of agreement.

A generalized linear mixed-effects model (GLME; Matlab, Mathworks) was used to evaluate cortisol level changes across training sessions and quantify the degree of reduction. The effects of the session and animal sex on the cortisol levels were tested using the following model: cortisolLevels~sessions × sex + 1|monkeyID. 

To assess whether the cortisol levels increased with the duration that the monkeys remained in the primate chair, a generalized linear mixed-effects model was fitted (GLME; Matlab, Mathworks). The effects of chair time and animal sex on cortisol levels were evaluated using the following model: cortisolLevels~chairTime × sex + 1|monkeyID. 

These analyses allowed us to assess the influence of training and chairing conditions on cortisol levels, providing insights into the stress responses of the subjects under different experimental conditions.

## 3. Results

### 3.1. Habituation and Animal Selection

After the trainer interacted with socially housed monkeys at the breeding facility, the first six monkeys were selected: three males: ‘Storm’, ‘Typhoon’, and ‘Thunder’; and three females: ‘Windy’, ‘Cloudy’, and ‘Christmas’. These individuals were chosen based on their confident approach toward the trainer, their acceptance of hand and syringe feeding, and their successful completion of the entire target training series. The selection process required five sessions of 10–15 min (Figure 1).

During the selection process, most male animals in social cages readily interacted with the trainer and accepted hand feeding from the first session. In contrast, only a few female monkeys were willing to accept hand feeding on the first day. This difference highlights potential gender-related variations in initial responsiveness to training, which could be attributed to behavioral differences or social dynamics within their cages.

In five cages clearly dominated by an alpha male, only the alpha males approached the trainer. This behavior highlights the influence of social hierarchy on individual participation in training activities. Conversely, in three cages, where primarily juvenile and subadult monkeys were housed, approximately one-third to half of the animals approached the trainer and accepted treats directly from the trainer’s hand. These observations suggest that social hierarchy and age composition within a group influence animal interaction and engagement in training.

### 3.2. Formal Training

**Figure 2:**
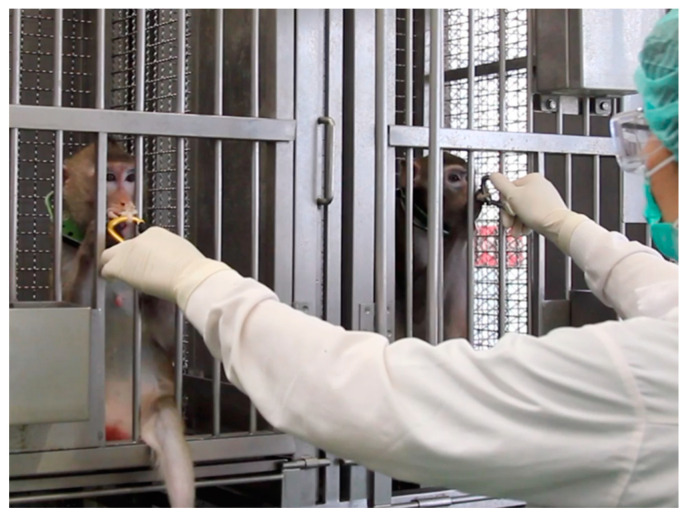
Baited-rope training.

**Baited-rope chewing:** five of the six monkeys chewed on the baited rope for 2 minutes, allowing voluntary saliva collection within three training sessions (Figure 2). However, one female monkey (‘Christmas’) only licked the rope to taste it but never chewed it. Despite continued attempts over three months, ‘Christmas’ persisted in licking the rope or untwisting the rope into small strings, which she then let fall to the cage floor.

**Figure 3:**
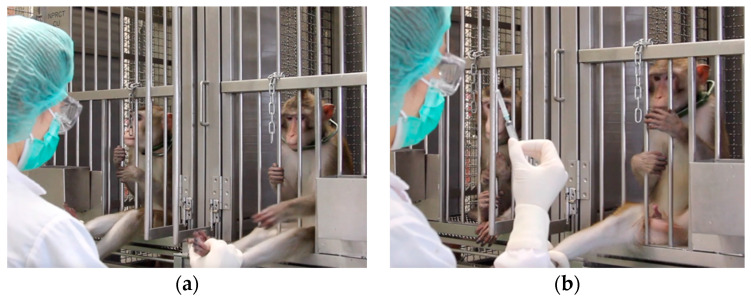
(**a**) Foot target and (**b**) needle training.

**Foot target and needle training in cage:** one male monkey, ‘Storm’, extended his leg and allowed the trainer to hold it within three training sessions. Two other male monkeys required the use of squeeze system, to gradually reduce the cage space and allow accidental foot touches, a few times before they learned to voluntarily extend their legs for a brief touch in exchange for treats. It took them up to 25 sessions before they let the trainer hold their leg, remained calm, and allowed the touch of a capped needle. Similarly, one female monkey, ‘Cloudy’, accidentally extended her leg during training then received a reward, leading to rapid success within seven sessions. The remaining two females required the squeeze system, achieving the leg-hold exercise within 22 sessions. After mastering the (capped) needle training (Figure 3), the animals were administered vitamin injections and then subjected to blood withdrawal using a needle and syringe. For the female monkeys, the blood samples were withdrawn from saphenous veins and then, on separate sessions and days, from femoral veins. In the male monkeys, blood withdrawal was only from the saphenous veins. The cages did not have appropriate-sized openings large enough for male monkeys to protrude their entire legs. Due to cage design limitations, in-cage femoral blood withdrawal was performed only in females.

**Pole-and-collar training:** Collar training took 4–16 sessions. The trainer successfully clipped the animals’ collars and guided them out of the cage to the weight scale or to the primate chair. Some monkeys, e.g., ‘Storm’ (M) and ‘Cloudy’ (F), were initially scared when having to leave their home cages, requiring a few sessions to habituate and calm down.

**Chair training:** The total number of training sessions per animal ranged from 1 to 45. The female monkeys were more compliant with entering the primate chair and calmly accepted the neck plate. One monkey (‘Christmas’) entered the chair from the first attempt and did not show resistance to the chairing process. The other two female monkeys took five sessions. On the other hand, the male monkeys were calm while sitting in the primate chair, but the neck-plate closing was the most challenging step, taking up to 45 sessions (for monkey ‘Storm’). The male monkeys tended to push the neck plate, stand up, and/or completely jump out of the chair when the trainer slid the neck plate; however, once familiar with the neck-plate closure, they remained entirely calm. After mastering chair training, blood withdrawal was smoothly performed in the monkey chair. All monkeys successfully completed chair training and in-chair single-blood-collection training.

**Serial blood collection:** After the animal mastered chair training, the chair time was gradually increased, only when the animal remained calm, until the chair time reached two hours. When the monkey could remain calm for the targeted duration, serial blood collection was performed using an IV catheter. All six monkeys managed to remain calm during the entire blood collection procedures within three sessions.

As previously reported [82,104,111], the interindividual variability in the number of sessions needed for each training step is shown in Table 1.

**Cortisol levels:** For single-blood-collection training, the cortisol levels decreased by an average of 87.51 ng/mL per training session (sessions: −87.51 ± 13.73 ng/mL; *t*-Stat = −6.3696; *p* < 0.001). There was no significant effect of animal sex on cortisol levels. Overall, the cortisol levels were substantially lower than the reference levels measured from the anesthetized reference (assayed from plasma aliquots during the facility’s annual health checks, when the animals were anesthetized; *n* = 43; 580.50 ± 2.88 ng/mL). In the trained animals, the cortisol levels could drop as low as 151.57 ng/mL when sampled in the cage and 135.53 ng/mL when sampled in the primate chair, levels that were never achievable with anesthetized sampling. 

**Figure 4:**
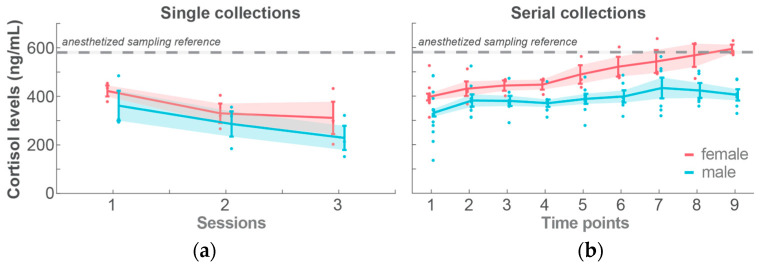
Cortisol hormone levels in (**a**) single-blood-collection training across sessions and (**b**) serial-blood-collection training across sampling time points.

Regarding serial-blood-collection training, although the animals did not show resistance during the chairing process, there was an increase in the cortisol levels over the sampling time points for both groups. In males, cortisol increased by 10.89 ± 2.47 ng/mL increase per 15-min interval (*t*-Stat = 4.4091; *p* < 0.001), while in females, the cortisol levels increased by 24.18 ± 3.48 ng/mL (*t*-Stat = 6.9514; *p* < 0.001). The female group exhibited a significantly greater increase than the male group (sex:timePoint: 13.288 ± 4.2663 ng/mL; *t*-Stat = 3.1146; *p* < 0.01; GLME: Figure 4b). The cortisol levels of the female monkeys approached the anesthetized reference after five time points, which was equivalent to an hour in the primate chair, while showing no behavioral resistance.

## 4. Discussion

The primary aim of this study was to assess the feasibility of a pilot behavioral training program for wild-sourced cynomolgus macaques, estimating the time investment required for each training exercise in this macaque population. Six cynomolgus monkeys were selected and trained to cooperate with standard husbandry and veterinary procedures, including target training, foot and needle training, pole-and-collar training, chair training, venipuncture, and serial blood collection using an IV catheter. All monkeys successfully completed the training program, except for one individual, who did not master baited-rope chewing for saliva collection. The monkeys completed needle training within 25 sessions, chair training within 45 sessions, and serial blood collection in a primate chair within three training sessions. 

Although cynomolgus monkeys are generally considered more despotic, fearful, and difficult to train compared to other macaque species [85,86,87], the training durations required to train the wild cynomolgus monkeys in the current study, e.g., a maximum of 45 sessions to sit in the primate chair, were shorter than previously reported, e.g., 85 sessions to chair-train rhesus macaques [111] and 6–7 months of venipuncture training in rhesus macaques and chimpanzees [104]. The relatively shorter training duration in this study may have resulted from the behavioral pre-screening and selection of monkeys based on temperament for the pilot training program. Without such temperament pre-screening, the training duration could have been longer. Future phases of the training project will include a broader range of animals, including those with less favorable temperaments, to further refine and improve the training protocol. 

Several areas for improvement in the pilot behavioral training program were identified. First, the baited-rope chewing exercise was not as intuitive for the monkeys as initially anticipated. The case of the monkey ‘Christmas’ highlights the need for a greater variety of soft and absorbent materials to encourage chewing, as well as more diverse liquid treats to motivate the animals. Therefore, future iterations of baited-rope training should aim to attract the animals within the first few sessions for more effective training. Second, the quadrant cages used in this study did not allow for convenient and safe in-cage femoral blood collection, particularly for the male monkeys. Blood sleeves have been shown to be effective for collecting blood from monkey arms [48,50,104]. However, as previously reported, macaques have small brachial veins, making arm blood collection challenging [104]. To address this, a ‘leg blood sleeve’ will be designed to provide access to the femoral and saphenous veins, substantially facilitating the in-cage blood collection. Third, initially, only behavioral criteria were used to assess training achievement, whereas the cortisol level measurements were intended to be part of this study’s results. Based on behavioral criteria, the male monkeys required approximately 10 times more sessions to complete chair training compared to the female monkeys. However, after concluding the training program, we conducted cortisol assays using the collected blood samples; we found that the sole behavioral criteria for training achievement turned out insufficient, especially for the female group, due to the high cortisol values during the late part of the chairing period. This suggests that the female monkeys may have tolerated stress without overt signs of resistance or aggression. Given that cortisol increases can result from both eustress and distress, the potential stress experienced by the monkeys warrants careful evaluation of both cortisol and behavioral criteria. Many aspects of sex differences in NHP behaviors have been reported [115], but fewer studies on sex differences in NHP stress responses and control are available. Further research on sex differences in NHP stress responses is needed to develop individualized stress management plans [36].

Upon further refinement, the cortisol levels should be included as a crucial measure of training achievement criteria to monitor animals’ stress levels during training and ensure they reduce to favorable levels at the conclusion of training exercises. If the cortisol levels remain high, the trainer may allow more adaptation time and/or adjust the training until all criteria are met. If the stress levels cannot be controlled, the animal should be excluded from the experiments to prevent conducting research with stressed animals that might tolerate undesirable procedures. Finally, the authors were aware of the advantages to using the ‘*direct load*’ technique for chairing monkeys [94,111], compared with the pole-and-collar method used in this study. The primate chairs used in the current study were custom-built, with an open design, allowing access to all parts of the monkey’s body. The pole-and-collar method in the current study was the only option for the existing chair design. The behavioral management team is planning on designing a primate chair that allows access to animals’ body parts via windows that can be flexibly closed and opened and, while at the same time allowing the direct loading of the animal into the chair.

Success in behavioral training requires both skills and time; behavioral reading and training skills are essential for trainers to effectively guide animals’ behaviors and ensure positive training experiences, while time and consistency are crucial for animals’ trust to build upon the accumulation of positive experiences; forcing or rushing are definitely counter-productive in training. Despite the time investment required for training, several well-known benefits of behavioral training after program initiation were observed: (a) the animals became more cooperative and exhibited positive behaviors toward the handling staff; (b) the animals showed excitement and anticipation toward training, such as vocalizing peeps and chirps when the trainer entered the room, trilling and twittering when food rewards were visible, and shaking behaviors when the trainer set up the training equipment [82]; and, finally, (c) the experiments that required trained animals performing cognitive tasks became possible.

With an actively ongoing breeding program, NPRCT-CU is working towards establishing sustained primate colonies. Although most research facilities rarely house wild-sourced primates and this will soon be the case for NPRCT-CU, the behavioral training program in this study can still benefit other captive primate settings, such as zoos, sanctuaries, shelters, and conservation sites, where wild animals live in captivity under human care [33,97]. Positive human–animal interaction and cooperative animals are essential to facilitate procedures and ensure safety for the care and veterinary personnel. Promoting animal welfare through behavioral training programs shall be integrated in skill training for future veterinarians, technicians, and care personnel, as well as be publicly promoted to increase public awareness of animal behavior and welfare [116], which can significantly benefit the society, especially in Thailand, where human–wildlife conflict remains a challenge.

## 5. Conclusions

Non-human primates (NHPs) are intelligent, social, and undomesticated, necessitating special attention for both their physical and psychological well-being. Ensuring their welfare is crucial not only for their health but also for maximizing their potential as highly valuable animal models. Overly stressed, fearful, or aggressive animals are less likely to succeed in training compared to those that are calm and cooperative. NPRCT-CU has initiated a behavioral training program for first-generation cynomolgus macaques, with all six selected monkeys successfully learning most of the training exercises within timeframes comparable to other studies. This behavioral training program, which incorporates temperament assessment, behavioral shaping plans, and stress hormone monitoring, represents a significant first step toward a larger population of well-trained and cooperative animals. Additionally, it serves as a foundation for developing more advanced training exercises and enhancing the skills of the staff involved. This study supports the use of behavioral training and related techniques in promoting NHP welfare. Cooperative behaviors in research animals are not only strong indicators of psychological health but also instrumental in facilitating both husbandry and research procedures. Last but not least, behavioral training paves the way for research that requires cooperative animals and their ability to learn and perform complex tasks, enabling studies that would be otherwise impossible without such cooperation.

## Figures and Tables

**Table 1:** Summary of the training sessions required for each training exercise.

Animal			Training Exercises (in Number of Sessions)				
ID	Sex	Age (year)	Saliva Collection	Foot Hold + Needle	Collar	Chair	IV Catheter
Storm (BTR085)	M	3	1	3	15	45	2
Typhoon (BTR098)	M	3	3	24	4	39	2
Thunder (BTR102)	M	3	3	25	8	34	3
Windy (BTR042)	F	5.5	2	22	8	5	2
Cloudy (BTR073)	F	6	3	7	10	5	1
Christmas (KS098)	F	3.5	*NA*	5	16	1	1
**Summary**			**1–3**	**3–25**	**4–16**	**1–45**	**1–3**

## Data Availability

The data presented in this study are available upon request from the corresponding author.

## Supplementary Materials

The following supporting information can be downloaded at: https://www.mdpi.com/article/10.3390/ani14162369/s1. Refs. [117,118] are cited in the supplementary file.
