# Supplementary material for: Behavioral Training in First-Generation Long-Tailed Macaques (Macaca fascicularis) for Improved Husbandry and Veterinary Procedures
Source: Animals (Basel). 2024 Aug 15;14(16):2369. doi: 10.3390/ani14162369 (PMC11350816; doi:10.3390/ani14162369)
Supplement: Supplementary file 1 [file animals-14-02369-s001.zip › animals-3031723-supplementary.pdf]

Supplementary methods

Shaping plans

1. Habituation: reinforcer bridging, hand feeding, and syringe feeding

a. Reinforcer bridging: *Primary and Secondary reinforcers*

The use of a clicker as a conditioned (secondary) reinforcer helps to mark desired behaviors and bridges the time gap between behaviors and delivery of food rewards (primary reinforcer). The clicker sound was initially a meaningless signal, but over time, when repeatedly paired with a primary reinforcer (i.e., food), it became a reinforcer [1–3]. Reinforcer bridging started early during the initial interaction with the animals. The animals were fed, and a clicker was sounded immediately before food was delivered to the animals [4,5]. The clicker-reward association was established for a few sessions until the animals showed positive responses to the clicker sound (e.g. lip-smacking, looking for reward). Once the association had been established, the clicks concurred with the desired behaviors, and the food reward arrived later. Once a strong association was established, the clicks (secondary reinforcer) could sometimes be used to 'reward' desired behaviors without offering the animals food rewards (primary reinforcer) [6–9].

As food items were a key motivator for monkeys [10], the trainer varied favored foods that were not part of the regular diet, e.g., sweet fruits (e.g. grapes, pineapples, bananas, apples, kiwis), nuts (e.g. peanuts, cashew nuts), seeds (e.g. sunflower, watermelon, pumpkin seeds), dried fruits (dates, raisins, cranberries, banana, mango, strawberries), and occasionally some highly favored snacks [4] (e.g. marshmallow, peanut butter crackers, milk tablets, cookies, gummies, and fruit juices). The occasional high-valued 'jackpots' allowed reinforcements with variable-ratio schedules that improved the response rate and reduced the extinction rate. Treats were small to prevent satiation and overfeeding (approximately ¼ of a small-sized grape, or ⅛ for a large-sized grape, per approximation; an average of 30 approximations per session).

b. Hand feeding (adapted from [4,6–8,11,12]:

Hand feeding is an important first step in establishing positive relationships with the animals and assessing animals' temperament in response to an approach of a new trainer. Some animals approached and accepted hand feed immediately (from step 1 directly to step 4 below), whereas others were more cautious and required successive approximations.

1. The animals were offered treats and were encouraged to take them from the trainer's hand.
2. If the animal did not approach and seemed nervous, the treats were placed on a horizontal cage bar in the animals' enclosure then the trainer stepped back from the enclosure front.
3. Once the animal had touched the treat, the trainer sounded the clicker device to bridge the primary and secondary reinforcers. The distance between the trainer and the animals was gradually decreased until the animals accepted hand feeding.
4. Once the animal accepted food from the trainer's hand, the trainer sounded the clicker and allowed the animal to receive the treat. The reward could be a large piece or a few pieces for the first few successes, after which it was gradually reduced. The hand feed was repeated at least 20 times until the animal was proficient.

Note: Risk of scratching: Certain individuals may grab during hand feeding and subsequent training steps. For physical protection, the trainer wore scratch-proof gloves in addition to latex gloves.

c. Syringe feeding

Syringe feeding is beneficial when treating animals with medications via the oral route. Some animals approached and drank from the syringe immediately (from step 1 directly to step 4), whereas others who might have had strong negative experiences with syringes showed clear fear responses toward the presentation of a syringe and needed successive approximations.

1. The animals were offered juice rewards using a syringe.
2. If the animal appeared nervous when using the syringe and regressed, the trainer released small drops of juice from the syringe on a horizontal cage bar and stepped back from the front of the enclosure.
3. The clicker device sounded when the animal drank the juice. The distance between the trainer and the animals was gradually decreased until the animals accepted the syringe feeding.

4. When the animal started drinking directly from the syringe; the trainer sounded the clicker and allowed the animal to drink from the syringe for a few seconds. The trainer gradually reduced the juice reward and repeated it at least 20 more times until the animal became proficient.

Note: Because the presentation of a syringe could be particularly aversive for some monkeys, the trainer used a positive reinforcer (food reward and clicker sound) in combination with the aversive stimulus (syringe), the animal was rewarded for remaining calm in the presence of the syringe, so that the fear response diminished over time (i.e. counter-conditioning; [7,8,11,13–15]).

2. Target and station training

The objective of target training was to instruct the animal to touch its body parts with a moveable object. In the current exercise, the trainer only requested hand touching.

Some animals immediately touched the target with their hand (from step 1 directly to step 4), whereas others were more anxious toward the target object (carabiner) and/or the trainer, so they needed successive approximations.

a. Hand target:

1. Animals were offered and encouraged to touch the target (either a carabiner or backhand-up cue as hand target) on command ('touch'; in Thai 'แตะ').
2. If the animal did not immediately approach and touch the target, the trainer clicked and treated if the animal looked at and/or moved toward the target.
3. Once the animal touched the target with any body parts, the trainer clicked and treated the animal for touching.
4. Once the animal touched the target with the hand, the trainer sounded the clicker and treated the animal. The reward could be a large piece or a few pieces for the first few successes, and then it gradually decreased.
5. After clicking, the trainer removed the target and presented it again as a new trial. The hand target was repeated at least 20 times until the animal was proficient.

Note: Some animals were cautious toward the target and did not touch the target for more than a week. The trainer performed additional steps between steps 2 and 3:

When the target was a carabiner,

(i) A food reward was placed on the target, and then the trainer clicked and rewarded the animal when it touched the reward on the target.

(ii) The food reward was held behind the target, and when the animal accidentally touched the target, the trainer clicked and rewarded.

(iii) The food reward was held at a certain distance that the animal could not reach while the target was held within the animal's reach. The trainer clicked and rewarded when the animal touched the target

In the case in which the target was the trainer's hand,

(i) The trainer placed the food reward on the palm with the hand completely opened, then clicked and rewarded when the animal took the reward.

(ii) The trainer placed the food reward on the palm and half-closed the hand (flexing the fingers slightly toward the wrist), then clicked and rewarded when the animal took the reward from the trainer's hand).

(iii) The food reward was placed on the trainer's palm, and the trainer closed the hand into a fist, the trainer clicked and rewarded the animal when the animal touched the trainer's hand to get the food reward.

b. Moving target

'Moving target' exercise can be useful for transporting and shifting compartments by placing a target at an aimed location. The animals typically learned the moving target exercise immediately after the hand target.

1. Once the animal had learned the target training, the trainer moved the target to a new location.
2. If the animal followed the target to the new location, the clicker was sounded and the animal was rewarded. The moving target was repeated at least 20 times until the animal was proficient.

c. Duration touch or hold target (adapted from [16])

'Hold target' exercise can be useful for station training, body inspection, weighing, etc.

1. When the animal touched the target, the trainer performed a delayed click; just briefly after the target touch.
2. Then, the delay was increased to 1 s and the trainer only clicked after the animal held the target until the aimed duration, then rewarded.

3. When the animal successfully held the target for five successive occasions, the duration of hold was increased, from 1 s to 3, 5, and 10 s, after each time five successive occasions had been reached.

Note: for some animals, continuous reinforcement while holding the target was beneficial for facilitating the initial few seconds of target holding. After the animals learned to hold for a few seconds, the continuous reinforcement was stopped and reward was delivered only after the intended duration.

d. Station training (adapted from [4,6,11])

1. Carabiners of different colors were used as stationing tools; each color was assigned to each monkey.
2. The trainer initially clipped the carabiners to the caging for the monkeys to investigate. Meanwhile, the trainer observed the animals' preferred locations so that the stationing locations were spontaneous and not too challenging for the monkeys (e.g. locations should minimize dominance interactions for subordinates).
3. When an animal (typically the dominant individuals would initiate) approached and touched a stationing tool for the first time, the animal was rewarded with a click, a treat, and a verbal cue with the animal's name. Subsequently, the given station was assigned to the monkey; this animal was only rewarded if he sat next to and touched his carabiner and was not rewarded for touching other carabiners.
4. This process was repeated for all other animals. If a monkey moved away from his station during training, the trainer would walk over to the monkey, point at him and say his name, then point at his station, and use the verbal cue of "station" (in Thai "เข้าที่").
5. The duration at which the monkey stayed at the station was gradually increased to 30 s.

Technical notes: Dealing with undesirable behaviors during habituation and target training (adapted from [4,6,11,13,17–20])

- *Unmotivated:* if the animal did not touch the target within a response time limit after target presentation (initially 10 s during habituation then shortened to 5 s later through the training program), the time was out, and the target was removed. The trainer waited until the animal appeared calm and focused and then gave the animal another chance. Sometimes, changing the treats could help motivate the animals.
- *Fear:* some animals were more fearful than others, especially during the early phase of training. Animals might refuse to cooperate and not accept food rewards. In this case, the trainer slowed down the training exercise and stepped back to the previously learned stage until the animals became comfortable moving forward with the training. Counter-conditioning, pairing fear-evoking stimuli with was used to alleviate fear responses.
- *Overenthusiastic:* An animal eagerly grasped a target without receiving a cue,
  - The trainer waited until the animal let go before immediately cuing "target" and clicked as the animal touched it again. Then, the trainer gradually shaped the inter-trial interval up to 30 s.
  - If the animal did not let go, the animal was fed away from the target. Once the animal was away from the target, we cued "target".
  - For certain overly motivated animals, it was helpful to specifically train the animal to release the target; the trainer said "release"; then, when the animal released the target, the clicker was sounded and the animal received treats. Training touch-hold-release together in sequence was particularly helpful for overenthusiastic animals to accustom them to the entire trial cycle.
- *Stagnated in training stages:* at certain training stages, the aimed behavior might not be trivial or too challenging for the animals, and some animals became confused and frustrated as the click/treat rate decreased. In this case, the trainer stepped back to the previous training step, in which the animals had acquired, to increase the click rate and then proceeded to the next challenging step that they needed to learn. Stepping back and forward between training stages was repeated multiple times until the animals became comfortable moving forward.
- *Aggression:* Alternate response technique was used to reduce aggression. If the aggression still persisted, short breaks and timeouts were used. We set five levels of reactions toward the animals' undesirable behaviors:
  - *Alternate response:* the trainer asked 'release' (in thai "ปล่อย"), in case of grabbing, or the trainer ignored the unwanted behavior by pausing the training for a few seconds and just looked at the ground to give the animal a chance to quit the aggressive behavior. The animal was reinforced for the next non-aggressive behavior exhibited.

- *Negative punishment (reward removal):* food rewards were kept out of sight (hidden in trainer's fist or lab coat pouch). The trainer remained with the animal, but training was paused for 5-15 seconds to give the animals a chance to calm down and relax (meanwhile the trainer may also gently say 'calm down' or 'relax'). If the animal managed to calm down, a reward was delivered and training resumed.
- *Time-out:* if the animal was still agitated, a timeout of 5-10 minutes was introduced. The trainer ignored negative behaviors, turned away from the animal, paused training, and may switch to interact with other animals during the timeout. The animal was rewarded for being calm when the trainer resumed training.
- *Call it a day:* if aggression or agitation still persisted after 2-3 timeouts, the trainer terminated the training session and resumed training the earliest on the following day.

3. Saliva collection using baited ropes

1. A sugar-coated rope was handed to the animal to investigate. When the animal started putting the rope in the mouth or chewing on the rope, the clicker was sounded, and the animal received a reward.
2. The rope was attached to the carabiner. Once the animal began chewing the attached baited rope, the clicker sounded.
3. The click was gradually delayed to increase the chewing time up to 2 min. After the desired chewing time, the clicker was sounded, and the animal was rewarded.

4. Presenting a leg for venipuncture

1. The animal was encouraged to move to the front of the cage using the "target" command.
2. The trainer used a backhand-down cue as the "foot" target. Any leg movement toward the target or accidental touching was rewarded.
3. Each time the animal's foot touched the target, the clicker was sounded and the animal was rewarded. This exercise was repeated until the animal reliably allowed the trainer to hold the leg.
   a. If the animal did not present the foot after a few weeks, with the help of the squeeze-back, the animal was confined in the front quarter of the cage. The animal was reassuringly talked to, touched, and rewarded. After a few minutes, the squeeze-back was pushed back and treats were offered. This exercise was repeated until the animal was relaxed and accepted the food reward with reduced cage space.
   b. Once the animal was comfortable being restricted with the squeeze-back, the trainer touched and groomed the animal's legs. When the animal was touched, the trainer clicked and treated the animal. This step was repeated until the animal stopped retracting its legs and accepted the food reward.
4. When the animal was comfortable having the trainer's hand wrap around its ankle, the trainer gently pulled the leg through the cage bars and held it for a few seconds. Then, the trainer clicked and rewarded the animal. This step was repeated until the animal remained calm during the leg hold.
5. The leg hold time was incremented from a few seconds up to 10 s. Continuous reinforcement of the juice bottle was provided during the leg hold time.
6. If the animal broke the position before the trainer released it, the continuous reinforcement was immediately removed and the desired behavior was simultaneously cued. Once the animal was in the desired position, the trainer immediately clicked and reinstated the continuous reward.
7. When the leg hold duration reached 10 s, the trainer rewarded the leg hold only intermittently to (1) allow the animal to become accustomed to the leg hold without reward, (2) reduce caloric intake from treats, and (3) delay satiation, allowing a longer hold.
8. Once the animal had achieved leg hold for up to 2 min, the trainer started to desensitize the animal to the implements needed during the blood collection procedure, including (1) the presence of veterinarians or technicians, (2) an electric razor, (3) alcohol swabs, (4) a syringe and capped needle, and, lastly, (5) a syringe and needle with real venipuncture. Success at each mini-step was clicked and rewarded.
9. The animals were considered to be reliably trained when they allowed venipuncture in three consecutive sessions without defensive reactions: attempts to grab the syringe, or exhibiting behaviors emblematic of fear, such as distress calls or attempts to pull their leg away to avoid syringe contact. Three venipuncture steps were sequentially conducted: vitamin injection (Selevit, Fercobsang, or Catosal) into the thigh muscle, blood withdrawal from the saphenous veins, and blood withdrawal from the femoral veins. Three consecutive successful sessions in each step led to the following step.

5. Collar-pole training

1. The animal was encouraged to move to the front of the cage using the "target" command.
2. A pole was introduced to the animal. The trainer clicked and rewarded the animal when the animal approached or touched the pole.
3. Once the animal was comfortable with the presence of the pole, the trainer touched the animal's collar using the pole, clicked, and treated the monkey for remaining calm.
   a. If the animal showed signs of fear or aggression as the pole approached, the trainer slowly backed the pole away until the animal appeared more comfortable, the animal was rewarded for getting calm, and the trainer approached with the pole again.
   b. If the animal did not voluntarily approach the pole after a few weeks of training, the squeeze mechanism was applied to reduce the cage space. Systematic desensitization and counter-conditioning to the squeeze mechanism (as described in Step 4.3 *Presenting a leg for venipuncture*) was conducted until the animal was in a position where the trainer needed to attach the pole, then the squeeze mechanism was released.
4. The pole-collar contact time was increased up to 5 s. Each time the animal remained calm while letting the pole come into contact with the collar, the trainer clicked and rewarded.
5. After the contact time reached 5 s for over 20 times, the trainer hooked the collar with the eyelet of the pole. If the animal became agitated, the trainer calmed the animal down, unhooked the animal, and then offered a bonus reward. The exercise was repeated until the collar was smoothly clipped and unclipped without resistance.
6. Finally, if the animal remained calm on the pole and could be guided out of the home cage to the weight scale or the primate chair without a fear response, the pole-collar training was considered successful.

6. Chair training

1. After the pole-and-collar training was completed, the animal was guided to a primate chair. Monkeys typically climbed up and sat on top of the chair, they were rewarded while sitting on the primate chair.
2. Once the animal was comfortable on the primate chair, the trainer slowly brought the animal down to sit on the sitting grid. The animal was only rewarded for sitting properly and remaining in position.
3. Then, the trainer (1) introduced the neck plate, (2) slowly slid the neck plate on the chair, (3) held it close to the animal's neck, and (4) fitted the neck plate loosely around the animal's neck. The animal was rewarded for remaining calm. At this stage, the animal typically broke the position by standing up or jumping out of the chair. The trainer progressed and regressed between these mini-steps until the animal accepted being neck-plated.
4. Once the animal was calm in the chair with the neck plate closed, the chair was wheeled around and/or out of their home enclosure to an adjacent area. While wheeling, the trainer continuously interacted with the animal and provided reinforcement for remaining calm.
5. The chair time was increased up to 15 min with intermittent rewards every 5 min.

7. Serial blood collection using an IV catheter

1. After the animal had mastered chair training, the chair time was incremented further from 15 min to 30, 60, 90, and 120 min with intermittent reward every 5-15 min (i.e. extended chair training). The chair time was increased only when the animal did not show distress or fear responses and accepted the treats during the intended duration of each increment.
2. During or after extended chair training, the animal was accustomed to the venipuncture process (as described in 4.8) followed by three trials of real venipuncture, on three separate days, using a syringe and needle. The animal was rewarded after each venipuncture.
3. When the animal remained calm in the chair for 2 h and accepted the venipuncture in the primate chair, the trainer started performing serial blood collection. An IV catheter was inserted into the animal's saphenous vein, and a 1-ml blood sample was collected every 15 min for 2 h (in total, 9 timepoints).
4. Any position break, leg retracting, whole-body spinning, or catheter pulling halted the procedure. The process was paused, and the animal was calmed down. If resistance persisted, the session was ended, followed by a one-week recovery period with the practices of behaviors the animal had already learned. Blood collection training was resumed in the following week.

5. The behavior was considered mastered when the animal allowed the collection of all nine blood samples while remaining calm.

References

1. Dorey, N.R.; Cox, D.J. Function Matters: A Review of Terminological Differences in Applied and Basic Clicker Training Research. *PeerJ* **2018**, *6*, e5621. https://doi.org/10.7717/peerj.5621.
2. Anderson, J.H.; Houghton, P. The Pole and Collar System: A Technique for Handling and Training Nonhuman Primates. *Lab. Anim.* **1983**, *12*, 47–49.
3. Skinner, B.F. How to Teach Animals. *Sci. Am.* **1951**, *185*, 26–29.
4. Prescott, M.J.; Bowell, V.A.; Buchanan-Smith, H.M. Training Laboratory-Housed Non-Human Primates, Part 2: Resources for Developing and Implementing Training Programmes. *Anim. Technol. Welf.* **2005**, *4*, 133–148.
5. McMillan, J.L.; Perlman, J.E.; Galvan, A.; Wichmann, T.; Bloomsmith, M.A. Refining the pole-and-collar method of restraint: Emphasizing the use of positive training techniques with rhesus macaques (Macaca mulatta). *J. Am. Assoc. Lab. Anim. Sci.* **2014**, *53*, 61–68.
6. Kemp, C.; Thatcher, H.; Farningham, D.; Witham, C.; MacLarnon, A.; Holmes, A.; Semple, S.; Bethell, E.J. A Protocol for Training Group-Housed Rhesus Macaques (Macaca Mulatta) to Cooperate with Husbandry and Research Procedures Using Positive Reinforcement. *Appl. Anim. Behav. Sci.* **2017**, *197*, 90–100. https://doi.org/10.1016/j.applanim.2017.08.006.
7. Laule, G.; Whittaker, M. Enhancing Nonhuman Primate Care and Welfare through the Use of Positive Reinforcement Training. *J. Appl. Anim. Welf. Sci.* **2007**, *10*, 31–38. https://doi.org/10.1080/10888700701277311.
8. Whittaker, M.; Laule, G. Training Techniques to Enhance the Care and Welfare of Nonhuman Primates. *Vet. Clin. N. Am. Exot. Anim. Pract.* **2012**, *15*, 445–454. https://doi.org/10.1016/j.cvex.2012.06.004.
9. Anderson, J.A.; Houghton, P. Training and handling nonhuman-primates in the captive and research environment. *Int. J. Primatol.* **1987**, *8*, 416.
10. Mason, S.; Premereur, E.; Pelekanos, V.; Emberton, A.; Honess, P.; Mitchell, A.S. Effective Chair Training Methods for Neuroscience Research Involving Rhesus Macaques (Macaca Mulatta). *J. Neurosci. Methods* **2019**, *317*, 82–93. https://doi.org/10.1016/j.jneumeth.2019.02.001.
11. Westlund, K. Training Laboratory Primates—Benefits and Techniques. *Primate Biol.* **2015**, *2*, 119–132. https://doi.org/10.5194/pb-2-119-2015.
12. Perlman, J.E.; Bloomsmith, M.A.; Whittaker, M.A.; McMillan, J.L.; Minier, D.E.; McCowan, B. Implementing Positive Reinforcement Animal Training Programs at Primate Laboratories. *Appl. Anim. Behav. Sci.* **2012**, *137*, 114–126. https://doi.org/10.1016/j.applanim.2011.11.003.
13. Nelson, F. The Role of Counterconditioning in the Extinction of Fear. *Am. Psychol.* **1961**, *16*, 468.
14. Keller, N.E.; Hennings, A.C.; Dunsmoor, J.E. Behavioral and Neural Processes in Counterconditioning: Past and Future Directions. *Behav. Res. Ther.* **2020**, *125*, 103532.
15. Meulders, A.; Karsdorp, P.A.; Claes, N.; Vlaeyen, J.W.S. Comparing Counterconditioning and Extinction as Methods to Reduce Fear of Movement-Related Pain. *J. Pain* **2015**, *16*, 1353–1365.
16. McKinley, J.; Buchanan-Smith, H.M.; Bassett, L.; Morris, K. Training common marmosets (Callithrix jacchus) to cooperate during routine laboratory procedures: Ease of training and time investment. *J. Appl. Anim. Welf. Sci.* **2003**, *6*, 209–220. https://doi.org/10.1207/s15327604jaws0603_06.
17. Reinhardt, V. Working with rather than against Macaques during Blood Collection. *J. Appl. Anim. Welf. Sci.* **2003**, *6*, 189–197. https://doi.org/10.1207/S15327604JAWS0603_04.
18. Reinhardt, V.; Reinhardt, A. *Environmental Enrichment and Refinement for Nonhuman Primates Kept in Research Laboratories: A Photographic Documentation and Literature Review*; Animal Welfare Institute: Washington, DC, USA, 2008; Volume 10, ISBN 9780938414926.
19. Minier, D.E.; Tatum, L.; Gottlieb, D.H.; Cameron, A.; Snarr, J.; Elliot, R.; Cook, A.; Elliot, K.; Banta, K.; Heagerty, A.; et al. Human-Directed Contra-Aggression Training Using Positive Reinforcement with Single and Multiple Trainers for Indoor-Housed Rhesus Macaques. *Appl. Anim. Behav. Sci.* **2011**, *132*, 178–186. https://doi.org/10.1016/j.applanim.2011.04.009.
20. Callen, E.J.; Boyd, T.L. Examination of a Backchaining/counterconditioning Process during the Extinction of Conditioned Fear. *Behav. Res. Ther.* **1990**, *28*, 261–271. https://doi.org/10.1016/0005-7967(90)90077-v.
